# Generation of transgenic rice with reduced content of major and novel high molecular weight allergens

**DOI:** 10.1186/s12284-014-0019-0

**Published:** 2014-08-29

**Authors:** Yuko Ogo, Yuhya Wakasa, Kana Hirano, Atsuo Urisu, Tsukasa Matsuda, Fumio Takaiwa

**Affiliations:** Transgenic Crop Research and Development Center, National Institute of Agrobiological Sciences, Kannondai 3-1-3, Tsukuba, 305-8604 Ibaraki, Japan; Graduate School of Bioagricultural Sciences, Nagoya University, Nagoya, 464-8601 Aichi, Japan; Department of Pediatrics, Fujita Health University, The Second Teaching Hospital, Nakagawa-ku, Nagoya, 454-8509 Aichi, Japan

**Keywords:** Food allergy, IgE antibody, Oryza sativa L, RNAi, Transgenic rice

## Abstract

**Background:**

Rice seed proteins contain antigens that provoke allergic responses in some individuals with food allergy, particularly in those with cereal allergy, and these antigens can elicit clinical symptoms such as eczema and dermatitis. We previously generated transgenic rice with reduced accumulation of the three major allergens, which dramatically reduced the level of IgE binding from patients’ sera. However, the transgenic rice still possesses allergenic reactivity. Recently, two globulin-like proteins were identified as candidates of novel high molecular weight (HMW) IgE-binding proteins that cause rice allergy.

**Results:**

We identified a glucosidase family encoded by four genes as novel HMW rice allergens based on IgE antibody reactivity from individuals with allergy to rice. To further reduce allergenicity, we generated transgenic rice with reduced accumulation of these HMW allergens. We crossed the rice with reduced HMW allergens and with reduced major allergens, and all major and HMW allergens were substantially reduced in the progeny of the crossed rice. Allergen suppression did not significantly alter accumulation patterns of seed storage proteins and protein folding enzymes. The sera of a portion of patients showed low IgE-binding to the crossed line, suggesting that the crossed line is effective for a portion of patients who are allergic to proteins other than major allergens.

**Conclusions:**

The transgenic rice with reduced levels of all major and HMW allergens is thought to be an option for a portion of allergy patients with hypersensitive responses to various kinds of rice allergens.

**Electronic supplementary material:**

The online version of this article (doi:10.1186/s12284-014-0019-0) contains supplementary material, which is available to authorized users.

## Background

Rice is a major cereal food consumed by more than half of the world population. The prevalence of IgE-mediated rice allergy is approximately 10% in atopic subjects. Symptoms of rice allergy include atopic dermatitis, eczema, and food-protein-induced enterocolitis syndrome (Hoffman [1975]; Shibasaki et al., [1979]; Ikezawa et al., [1992]; Sicherer et al. [1998]; Uchio et al., [1998]; Mehr et al., [2009]). Multiple rice seed proteins are responsible for rice allergy (Urisu et al., [1991]). Among them, α-globulin (26 kDa), β-glyoxalase I (33 kDa), and α-amylase/trypsin inhibitor (14–16 kDa) were identified as major rice allergens based on recognition by IgE from individuals with food allergy (Alvarez et al., [1995]; Limas et al., [1990]; Usui et al., [2001]; Matsuda et al., [2006]). The 14–16 kDa α-amylase/trypsin inhibitors constitute a multigene family, whereas the 26 and 33 kDa allergens are encoded by single-copy genes. These allergens strongly react with IgE antibody in sera from many individuals with rice allergy, and caused eczematous and atopic dermatitis (Urisu et al., [1991]).

Avoidance of food containing allergens is one of the most important therapeutic strategies for those with food allergy. Allergens have been removed from rice by several processing technologies such as enzymatic digestion, alkaline hydrolysis, and high hydrostatic pressure (Watanabe et al., [1990a], [1990b]; Kato et al., [2000]). Some of these processes have been commercialized to produce low-allergen rice in Japan; however, the taste quality of processed rice is reduced by these chemical, enzymatic, or physical treatments, and the treatments are costly. Therefore, it is important to develop a cost-effective means to produce low-allergen (hypo-allergenic) rice with good taste. We previously generated hypo-allergenic transgenic rice, in which the levels of major seed allergen genes (26 kDa, 33 kDa, and 14–16 kDa allergens) were suppressed (Wakasa et al., [2011a]). To suppress the major allergen levels in rice grains, we first found a mutant in the Koshihikari background that lacked the 26 kDa allergen (GbN-1). Then, 33 kDa and 14–16 kDa allergen levels were suppressed by RNA interference (RNAi) using GbN-1 as a host rice. In the transgenic line, the content of the three major allergens was remarkably reduced to a very faint level. IgE binding of patients’ sera to the transgenic rice seed with reduced levels of major allergens was substantially lower compared with that of non-transgenic (NT) Koshihikari (Wakasa et al., [2011a]). However, some individuals retained allergenic reactivity to the transgenic rice. These results indicate that additional allergens are present in rice, which must be removed for the optimum health of individuals with a wide variety of allergies to rice.

Several major rice allergens were identified in previous studies. Urisu et al. ([1991]) showed that IgE from patients with rice allergy detected several allergenic proteins with high molecular weight (HMW), which have not yet been identified. Two globulin-like proteins of 52 kDa and 63 kDa, which correspond to the HMW allergenic proteins, have recently been identified as novel IgE-binding proteins. These globulin-like proteins are candidates for rice allergens based on recognition by IgE from patients with rice allergy (Satoh et al., [2011]). The 52 kDa and 63 kDa proteins are strongly expressed in rice seed, and are thought to be major causes of rice allergy. Here, we identified a multigene glucosidase family as novel IgE-binding HMW proteins, which are also candidates for rice allergens. We suppressed the levels of these HMW allergens by RNAi and crossed the RNAi rice with the transgenic rice with reduced levels of the major allergens. The allergen-reduced transgenic rice is a promising candidate for generating hypo-allergenic rice.

## Results and discussion

### Identification of HMW rice allergens

**Figure 1:**
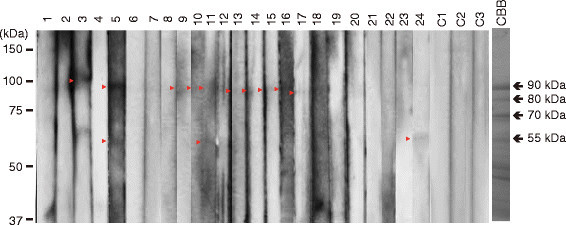
**Western blotting of albumin-globulin fraction protein using sera of patients with rice allergy.** Lanes 1 to 24, sera from patients; Lanes C1 to C3, sera from healthy volunteers. Red arrow heads indicate positive bands detected by sera. Bands indicated by arrows (90 kDa and 55 kDa) are the proteins subjected to peptide identification by MALDI-TOF MS/MS.

To identify HMW candidate proteins for rice allergens that have not been previously analyzed, we performed western blot with serum IgE from 24 rice-positive patients using gels with low acrylamide concentrations. The albumin-globulin rice extract was subjected to 1D SDS-PAGE. Two proteins of approximately 90 kDa and 55 kDa, which were reactive to IgE from several patients with rice allergy (Urisu et al., 1999), were detected (Figure 1). These proteins were not detected with the control sera. To identify these proteins, the two bands were cut from Coomassie brilliant blue-stained gels, in-gel digested with trypsin, and subjected to protein identification by MALDI-TOF-MS/MS. The 90 kDa protein was identified as an α-glucosidase (ONG1; Os06g0675700) (Table 1). Database searches identified two ONG1 homologs, ONG2&3 (Os06g0676700) and ONG4 (Os01g0130400), whose amino acid similarities to ONG1 are 97% and 90%, respectively. ONG1, ONG2&3, and ONG4 were reported as proteins extracted from rice seeds (Nakai et al., [2007]). The 55 kDa protein was identified as protein disulfide isomerase (PDI) 1 (OsPDIL1;1; Os11g0199200) (Table 1). PDI is essential for protein folding in the ER lumen, and is involved in catalysis of disulfide bond formation (reduction or isomerization) and in assisting polypeptide folding (Wilkinson and Gilbert, [2004]; Gruber et al., [2006]). Rice contains 19 PDI-like genes (Houston et al., [2005]), and OsPDIL1; 1 is one of the major rice PDI proteins. Failure to express OsPDIL1;1 in rice results in a floury endosperm and an endoplasmic reticulum stress response in rice (Satoh-Cruz et al., [2010]; Han et al., [2012]).

**Table 1:** **Identification of IgE-binding rice proteins by MALDI-TOF MS/MS**

Name	Locus ID	MW (kDa)	MS		MS/MS	
			Sequence coverage (%)	Matched peptide	Sequence coverage (%)	Matched peptide
ONG1	Os06g0675700	90	27	15	14	7
OsPDIL1;1	Os11g0199200	55	23	12	10	4

### Generation of transgenic rice with reduced levels of the HMW allergens

**Figure 2:**
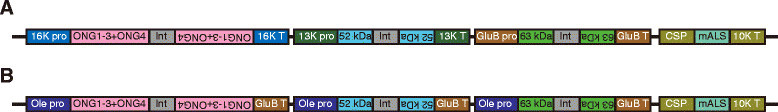
**Schematic representation of the binary vector T-DNA. A.** Endosperm-specific suppression. **B.** Endosperm- and embryo-specific suppression. 16 K pro, *16 kDa prolamin* promoter; 16 K T, *16 kDa prolamin* terminator; 13 K pro, *13 kDa prolamin* promoter; 13 K T, *13 kDa prolamin* terminator; GluB pro, *glutelin B-1* promoter; GluB T, *glutelin B-1* terminator; Ole pro, *18 kDa oleosin* promoter.

To reduce rice allergenicity, we suppress the HMW allergens identified above using RNAi. However, OsPDIL1; 1 plays an important role in seed storage protein accumulation, and knockdown of OsPDIL1; 1 severely disrupts seed quality. OsPDIL1; 1 was not selected for suppression in the present experiment. ONG1 was selected for suppression, because no studies have reported that ONG knockdown significantly affects seed quality. In addition to ONG1, we suppressed ONG2&3 and ONG4 because they are highly homologous to ONG1 and accumulate in seeds. Satoh et al. ([2011]) recently identified two globulin-like proteins of 52 kDa and 63 kDa as HMW IgE-binding proteins that are candidates for rice allergens. In this study, we also suppress the 52 kDa and 63 kDa globulins by RNAi. The 63 kDa globulin, ONG2&3, and ONG4 are expressed in embryo and endosperm, whereas the 52 kDa globulin and ONG1 are expressed primarily in endosperm (Additional file 1: Figure S1). We use several endosperm-specific promoters, which confer expression throughout the whole endosperm, and *18 kDa oleosin* promoter, which confers embryo- and endosperm-specific expression but does not confer expression at the inner side of endosperm. To generate transgenic rice in which the HMW allergens were strongly suppressed throughout the endosperm, we used the endosperm-specific promoters (*13 kDa prolamin*, *glutelin B-1*, and *16 kDa prolamin* promoters) to express RNAi constructs of the HMW allergens. To prevent possible contamination of the HMW allergens from embryo, we used the *18 kDa oleosin* promoter to express RNAi constructs of the HMW allergens (Figure 2). A mutated acetolactate synthase gene (mALS) under callus-specific promoter (CSP) was used as a selectable marker gene, which was acceptable for commercial cultivation because they were derived from the rice genome and selection was restricted to the callus stage. These binary vectors were introduced into Koshihikari, which is the most popular cultivated elite variety in Japan due to its excellent taste. We generated 30 transgenic lines with endosperm-specific suppression, and endosperm- and embryo-specific suppression of the HMW allergens each.

### Western blot of 52 kDa and 63 kDa globulins

**Figure 3:**
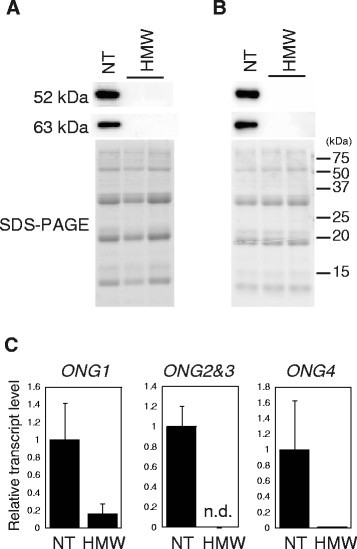
**Generation of rice lines with reduced levels of the HMW allergens. A**. Western blot of 52 kDa and 63 kDa globulins in transgenic rice with endosperm-specific suppression of the HMW allergens. **B**. Western blot of 52 kDa and 63 kDa globulins in transgenic rice with endosperm- and embryo-specific suppression of the HMW allergens. **C**. Real-time PCR of *ONG1*, *ONG2&3*, and *ONG4* using transgenic rice with endosperm- and embryo-specific suppression of the HMW allergens. NT, non-transgenic Koshihikari; HMW, transgenic rice with reduced levels of the HMW allergens.

We generated antibodies against the 52 kDa and 63 kDa globulins and investigated protein expression patterns by western blotting. Protein was extracted from mature seeds without embryos of transgenic rice with endosperm-specific suppression of the HMW allergens. Both 52 kDa and 63 kDa globulins were strongly repressed in endosperm from several lines (Figure 3A). Protein was extracted from mature seeds including embryos of transgenic rice with endosperm- and embryo-specific suppression of the HMW allergens. Both 52 kDa and 63 kDa globulins were suppressed in whole seeds including embryos in several lines (Figure 3B). The RNAi constructs driven by the *18 kDa oleosin* promoter substantially suppressed 52 kDa and 63 kDa globulins in whole seeds. We used transgenic rice with endosperm- and embryo-specific suppression of the HMW allergens for further studies.

### Real-time PCR of *ONG1*, *ONG2&3*, and *ONG4*

We attempted to generate antibodies against ONG1, ONG2&3, and ONG4; however, strongly reactive antibodies to ONGs were not produced. Therefore, mRNA expression of *ONG1*, *ONG2&3*, and *ONG4* in transgenic rice seeds was investigated by real-time PCR. We extracted total RNA from transgenic rice seeds with endosperm- and embryo-specific suppression of the HMW allergens and performed real-time PCR. The mRNA levels of *ONG1*, *ONG2&3*, and *ONG4* were substantially reduced in a few lines (Figure 3C). According to the Western blot of 52 kDa and 63 kDa globulins and real-time PCR of *ONGs*, we identified a line in which the levels of 52 kDa globulin, 63 kDa globulin, *ONG1*, *ONG2&3*, and *ONG4* were substantially suppressed in endosperm and embryo.

### Crossing transgenic rice lines with reduced levels of major and HMW allergens

We crossed transgenic rice lines with reduced levels of the HMW allergens in endosperm and embryo, and transgenic lines with reduced levels of the major allergens. In the lines with reduced levels of the major allergens, the 33 kDa and the 14–16 kDa protein were suppressed by RNAi, whereas the 26 kDa protein was knocked out by γ-ray irradiation mutagenesis (Iida et al., [1998]; Wakasa et al., 2011). Suppression of the 26 kDa protein was only observed in the recessive homozygote of the GbN-1 mutation; therefore, F_2_ seeds were analyzed. The F_2_ seeds were cut in half, and protein was extracted from the half without the embryo. The 26 kDa protein was suppressed in 90 of 397 F_2_ seeds (23% of the total seeds), and the 52 kDa globulin was suppressed in 220 of 397 F_2_ seeds (55% of the total seeds) (Table 2). We identified 55 of 397 F_2_ seeds (14% of the total seeds) in which both the 26 kDa and the 52 kDa proteins were suppressed. Of these 55 F_2_ seeds, the 33 kDa, the 14–16 kDa, and the 63 kDa protein levels were substantially suppressed in 32, 32, and 48 F_2_ seeds, respectively. We identified 24 F_2_ seeds (6% of the total seeds) in which the 26 kDa, the 33 kDa, the 14–16 kDa, the 52 kDa, and the 63 kDa protein levels were substantially suppressed (Table 2).

**Table 2:** **Target gene suppression efficiency in transgenic rice lines**

	Total F2 seed	26 kDa	52 kDa	26 & 52 kDa	Among both 26 kDa and 52 kDa suppressed seeds,			All
					33 kDa	14-16 kDa	63 kDa	
Number/investigated seeds	397	90/397	220/397	55/397	32/55	32/55	48/55	24/397
%	100	23	55	14	–	–	–	6

% indicates percentage of the seed number to the total F_2_ seed number (397). 26 kDa, 52 kDa, and 26 & 52 kDa indicate the number of the transgenic seeds with reduced level of the 26 kDa, the 52 kDa, and both the 26 kDa and the 52 kDa allergens, respectively. 33 kDa, 14–16 kDa and 63 kDa indicate the number of the seeds with reduced levels of the 33 kDa, the 14–16 kDa and the 63 kDa allergens, respectively, among the transgenic seeds with reduced levels of both the 26 kDa and the 52 kDa allergens (55 seeds). All indicates the number of transgenic seeds with reduced levels of all the major and the HMW allergens.

**Figure 4:**
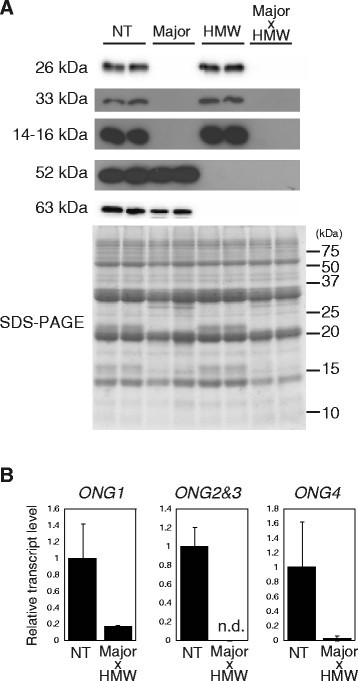
**Crossing of transgenic rice lines with reduced levels of the major and the HMW allergens. A**. Western blot of the major allergens (26 kDa, 33 kDa, and 14–16 kDa) and the HMW allergens (52 kDa and 63 kDa allergens). **B**. Real-time PCR of *ONG1*, *ONG2&3*, and *ONG4*. NT, non-transgenic Koshihikari; Major, the rice lines with reduced levels of major allergens; HMW, the rice lines with reduced levels of the HMW allergens (endosperm- and embryo-specific suppression); Major x HMW, the rice lines with reduced levels of the major and the HMW allergens.

The remaining F_2_ seed halves containing embryos were grown and F_3_ seeds were harvested. We obtained several F_3_ lines in which the 26 kDa, the 33 kDa, the 14–16 kDa, the 52 kDa, and the 63 kDa allergen levels were substantially suppressed (Figure 4A). Total RNA was extracted from these F_3_ seeds and real-time PCR of *ONG* genes was performed. We identified F_3_ seeds in which expression of *ONG1*, *ONG2&3*, and *ONG4* was substantially suppressed. We obtained a few F_3_ lines in which the contents of all major and HMW allergens were reduced. These lines were named as Major x HMW rice.

### Allergenic potential of the rice lines with reduced levels of the major and HMW allergens

**Figure 5:**
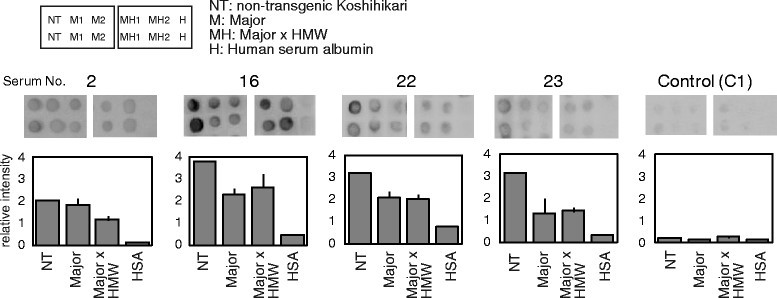
**IgE binding to seed extracts of rice lines with reduced levels of the major and the HMW allergens.** Typical dot-blotting images of four representative serum specimens are shown together with immuno-dot signal intensities quantified using NIH Image-J. Seed protein extracts were spotted in duplicate on the membrane from the non-transgenic Koshihikari (NT), two lines (M1 and M2) of transgenic rice with reduced content of the major allergens (Major), and two lines (MH1 and MH2) of Major x HMW lines. Human serum albumin (H, HSA) was used as a negative antigen control. A control serum specimen with a low content of IgE to rice seed proteins was also analyzed for comparison. The dot intensities to Major and Major x HMW are shown as mean ± SD of four dots, while those of NT and HSA are mean of two dots. The serum numbers correspond to those shown in Figure 1.

In the previous study, IgE binding of protein extracts from the line with reduced levels of the three major allergens was analyzed by immuno-dot-blotting analysis with sera from 15 patients (Wakasa et al., [2011a]). The results showed that IgE reactivity of sera from 9 patients was dramatically reduced (>90%), whereas that of 5 patients was not substantially decreased, suggesting that a significant number of patients were allergic to proteins other than the three major allergens. Major x HMW rice may be an option for allergy patients with hypersensitive responses to various kinds of rice allergens. To estimate allergenic potential of the rice seeds of these lines, IgE binding of seed protein extracts from two Major x HMW lines was analyzed using immuno-dot blotting using serum specimens from 10 allergic patients with relatively high levels of serum IgE to rice seed proteins. Typical IgE dot-blotting images of four representative serum specimens are shown in Figure 5. Only two serum specimens including serum 2 showed lower IgE-binding to Major x HMW lines than to the lines with reduced contents of the major allergens, while the others showed IgE binding to Major x HMW lines almost equally to the Major lines or tended to give even slightly high IgE-binding (Figure 5). These results suggested that Major x HMW rice is effective for a portion of the patients who are allergic to proteins other than major allergens, although it may not be effective for the other patients. Thus, future practical utilization of the hypoallergenic Major x HMW lines would become more effective in combination with personal clinical tests for patients’ serum IgE specificity to rice allergen components.

### Seed characteristics in rice lines with reduced levels of the major and HMW allergens

**Figure 6:**
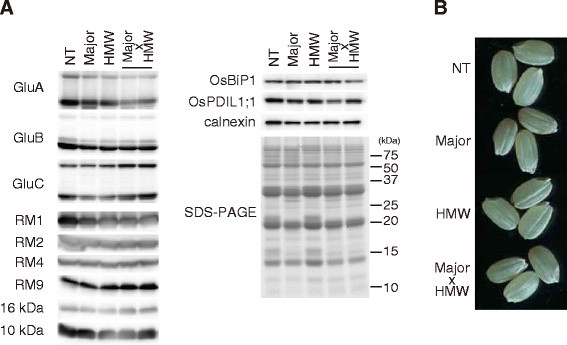
**Characterization of rice lines with reduced levels of the major and the HMW allergens. A.** Western blot of seed storage proteins and proteins involved in protein folding. **B.** Seed phenotype. NT, non-transgenic Koshihikari; Major, the rice lines with reduced levels of the major allergens; HMW, the rice lines with reduced levels of the HMW allergens (endosperm and embryo-specific suppression); Major x HMW, the rice lines with reduced levels of the major and the HMW allergens.

We investigated the accumulation of seed storage proteins in mature seeds. In transgenic rice lines with reduced levels of the major allergens, with reduced levels of the HMW allergens, and with reduced levels of both the major and the HMW allergens, glutelins (GluA, GluB, GluC) and prolamins (RM1, RM2, RM4, RM9, 16 kDa prolamin, 10 kDa prolamin) accumulated to the same levels as observed in NT Koshihikari (Figure 6A). Reduction of the major and the HMW allergen levels did not significantly alter the accumulation of most seed storage proteins, which may be explained by the low expression level of these allergens (<5% by weight of total seed proteins). There was no significant difference in the amounts of OsBiP1, OsPDIL1; 1 and calnexin among NT Koshihikari and transgenic rice lines (Figure 6A). OsBiP1, OsPDIL1; 1 and calnexin levels are sensitive to perturbations of the protein folding and transport system in the ER lumen (Oono et al., [2010]; Wakasa et al., [2011b]). However, the observation that OsBiP1, OsPDIL1; 1 and calnexin protein levels did not significantly differ among all lines suggests that the ER protein folding and transport system was not disrupted in the transgenic rice seed cells.

The seed morphology of the transgenic lines was investigated (Figure 6B). No significant difference in seed length, seed thickness, and protein content were observed among all lines (Table 3). The seed weights of the lines with reduced levels of the major allergens and Major x HMW lines were 14–15 mg/seed, whereas that of NT Koshihikari and the lines with reduced levels of the HMW allergens were 17–18 mg/seed (Table 3). Seed width of the lines with reduced levels of the major allergens and Major x HMW lines was less than those of NT Koshihikari and the lines with reduced levels of the HMW allergens (Table 3). The reduction of seed weight and seed width is thought to be inherited from GbN-1. Wakasa et al. ([2011a]) reported that seed weights of GbN-1 and the lines with reduced levels of the major allergens were slightly lower than NT Koshihikari. GbN-1 is sensitive to adverse conditions and shows reduced growth, yield, and seed weight under unfavorable conditions (unpublished data). Conversely, GbN-1 growth is not delayed and the seeds are maintained in good quality with favorable conditions such as a large field and good insolation conditions. In the present study, the lines with reduced levels of the major allergens and Major x HMW lines showed strong GbN-1 characteristics because rice plants were grown in small pots, in which rice could not set their roots deep enough. Backcross to NT Kosihikari is effective to diminish these undesirable GbN-1 phenotypes and improve the seed quality of Major x HMW lines.

**Table 3:** **Seed characteristics in the transgenic rice lines**

Name	Length (mm)	Width (mm)	Thickness (mm)	Weight (mg/seed)	Protein content (%)
NT	4.71 ± 0.24	2.65 ± 0.09	1.84 ± 0.04	17.9 ± 0.5	13.5 ± 0.6
Major	4.65 ± 0.18	2.27 ± 0.30	1.75 ± 0.09	14.0 ± 0.7	14.9 ± 0.9
HMW	4.68 ± 0.08	2.68 ± 0.11	1.78 ± 0.05	16.9 ± 0.6	13.6 ± 1.2
Major x HMW	4.75 ± 0.21	2.44 ± 0.20	1.78 ± 0.04	15.0 ± 0.6	14.5 ± 1.3

Seed length, width, thickness, and weight, *n* = 8; total protein content, *n* = 3. Values represent means ± standard deviation. NT, non-transgenic Koshihikari; Major, the rice lines with reduced levels of the major allergens; HMW, the rice lines with reduced levels of the HMW allergens (endosperm and embryo-specific suppression); Major x HMW, the rice lines with reduced levels of the major and the HMW allergens.

Once the safety evaluation of the transgenic rice is performed, Major x HMW lines can be utilized by individuals with a rice allergy whose serum IgE recognizes these allergens. The transgenic rice approach has several advantages over conventional hypo-allergenic rice produced by processing with enzymatic and physical treatments. Our hypo-allergenic rice was generated using the highly edible Koshihikari variety, and the transgenic product is cost-effective. The seed quality of Major x HMW lines can be improved by backcrossing with Koshihikari. Major x HMW lines are thought to be acceptable as a hypo-allergenic rice for a portion of patients with rice allergies.

## Conclusions

We identified a glucosidase family as novel rice HMW allergens. We successfully generated transgenic rice with reduced content of the major and the HMW allergens, which showed reduced IgE binding to sera of a portion of rice allergy patients who are allergic to proteins other than the major allergens. These transgenic rice are thought to be an option for a portion of allergy patients with hypersensitive responses to various kinds of rice allergens.

## Methods

### SDS-PAGE and Western blot for identification of HMW allergens

Seed proteins (the albumin-globulin fraction) were extracted from rice seeds (*Oryza sativa* cv. Koshihikari), as described previously (Wakasa et al., [2011a]). The extracted proteins were separated by SDS-PAGE (10% acrylamide), followed by Western blot using the serum IgE from individual patients, as described previously (Usui et al., [2001]; Hirano et al., [2013]). Protein bands bound with IgE were immunologically detected with peroxidase-labeled anti-human IgE antibody (Nordic immunological Laboratories, Susteren, Netherlands) and a chemiluminescence detection kit (GE Healthcare Biosciences, Piscataway, NJ, USA).

### Patient serum samples

Blood was collected from patients with suspected allergic disorders including food allergy under medical treatment at the Hospital of Fujita Health University. Informed consent was obtained from the patients or guardians of infants or child subjects. Serum specimens were prepared from fresh blood and kept as 50% glycerol mixture at −30°C until use. This study using patient serum specimens was approved by the Ethics Committee of Fujita Health University School of Medicine. Twenty-four serum specimens with relatively high IgE-binding to the rice seed albumin/globulin fraction were selected as rice-positive specimens, whereas three specimens with relatively low IgE-binding were selected as control specimens.

### Protein identification by MS/MS analysis

Protein bands detected by Coomassie brilliant blue staining were excised, in-gel digested with trypsin, and extracted according to the manufacturer’s instructions. MS and MS/MS (tandem MS) analyses were performed using a MALDI–TOF/TOF mass spectrometer (4700 Proteomics Analyzer; Applied Biosystems, CA, USA), as described previously (Okumura et al., [2004]). The obtained MS and MS/MS data were analyzed using Mascot Daemon data analysis software (Matrix Science; http://www.matrixscience.com/).

### Immuno-dot-blot analysis of rice seed proteins for human IgE

IgE binding of patients’ IgE was analyzed by dot-blotting for limited amounts of serum specimens as described previously (Wakasa et al., [2011a]). Briefly, an aliquot of each rice seed extract, which was equivalent to 100 μg of milled rice seeds, was spotted on a small piece of nitrocellulose membrane, blocked with gelatin solution, and then incubated with 100-fold diluted human serum specimen. The IgE bound to the rice proteins on the membrane was detected using HRP-labeled secondary antibody and a chemiluminescence HRP substrate, and the chemiluminescence intensity was quantified using NIH Image-J.

### Plant material and growth conditions

Rice plants (*Oryza sativa* cv. Koshihikari) were grown at 25°C/20°C (12 h/12 h day/night cycles) in 12 cm diameter pots containing a commercial soil mixture (Bonsol No. 1; Sumitomo Chemicals, Osaka, Japan) with 14-14-14 chemical fertilizer. The host of the line with reduced levels of major allergens is a seed storage protein mutant named GbN-1 (cv. Koshihikari background), which lack the 26 kDa allergen (Iida et al., [1998]).

### Vector construction

Binary vectors were constructed for *Agrobacterium*-mediated transformation using the MultiSite Gateway LR clonase reaction (Invitrogen), as described previously (Wakasa et al., [2006]). Briefly, gene cassettes consisting of an endosperm-specific promoter [*16 kDa prolamin* promoter (0.93 kb), *13 kDa prolamin* promoter (1.23 kb), and *GluB-1* promoter (2.4 kb)], the inverted repeat structure of sense and antisense fragments from the coding region of *ONG1-4*, *52 kDa globulin* (0.76 kb), or *63 kDa globulin* (0.92 kb) separated by the rice *oryzasin1* intron (0.99 kb) (Asakura et al., [1995]; Kuroda et al., [2010]), and a terminator [*16 kDa prolamin* (0.62 kb) terminator, *13 kDa prolamin* terminator (0.18 kb), or *GluB-1* terminator (0.6 kb)] were inserted into the Gateway entry clones pKS221 MCS, pKS 4–1 MCS and pKS 2–3 MCS. The homologous region of *ONG1* and *ONG2&3* (0.72 kb), and part of *ONG4* (0.7 kb) were fused and used as an inverted repeat structure of sense and antisense fragments of *ONGs*. These gene cassettes were introduced into pCSP mALS 43GW using MultiSite Gateway LR Clonase II Plus Enzyme Mix (Invitrogen) (Figure 2). The binary vector plasmids were introduced into the Koshihikari cv. via *Agrobacterium*-mediated transformation, as described previously (Wakasa et al., [2007]).

### Protein extraction and Western blot analysis of the reduced allergen rice

Total protein extraction and Western blot were performed as described previously (Wakasa et al., [2011a]). Antibodies to 26 kDa, 33 kDa, and 14–16 kDa allergens, glutelins (GluA, GluB, and GluC), RM1, RM2, RM4, RM9, 16 kDa prolamin, 10 kDa prolamin, OsBiP1, OsPDIL1;1, and calnexin were previously prepared in our laboratory (Takagi et al., [2006], Yasuda et al., [2009]; Wakasa et al., [2011a], [2011b]). The MH_2_-RRGEREEEDERRRHG –OH and MH_2_-SGEDRRRETSLRRC -OH peptides derived from 52 kDa (Os03g0793700) and 63 kDa (Os03g0663800) allergens, respectively, were synthesized and used to raise anti-52 and 63 kDa allergens polyclonal antibody in a rabbit (Scrum Inc., Tokyo, Japan). Seed protein content was measured by an RC DC protein assay kit (Bio-Rad), as described in the manufacturer’s protocol.

### RNA preparation and real-time PCR

Total RNA was prepared from mature seeds of NT and transgenic rice plants, as described previously (Yasuda et al., [2005]). Then, cDNA was synthesized from 500 ng of total RNA using ReverTra Ace qPCR RT Master Mix with gDNA Remover (TOYOBO, Osaka, Japan), according to the manufacturer’s instructions. Real-time PCR was performed using SYBR Premix Ex Taq (TaKaRa; http://www.takara-bio.com). Primer pairs for amplification were as follows: for *ONG1*, (5’-ACTCCATCAACACCATGCTC-3’ and 5’- CGGTGCCGATCGCCGAGTGA-3’); for *ONG2&3*, (5’-GGCCATTAGCATCGCAAGCT-3’ and 5’-CCTCATCCACCAGGAATGCC-3’); and for *ONG4*, (5’-GGATCGACGAGGTGAGGAGG-3’ and 5’-TCCCACCTGGTGTTCGTCAG-3’). *Ubiquitin* (*Os06g0681400*) was amplified as an internal reference using the primer set (5’-GTGGTGGCCAGTAAGTCCTC-3’ and 5’-GGACACAATGATTAGGGATCA-3’).

### Application of RiceXpro

To investigate expression levels of the HMW rice allergens in endosperm and embryo (Additional file 1: Figure S1), we utilized the low expression-level data of reproductive organs (inflorescence, anther, pistil, ovary, embryo, and endosperm) of RiceXpro (Sato et al., [2011]). The signal intensity was normalized by the 75th percentile. The signal intensity represented in the bar graphs shows the average of three biological replicates ± SD. The signal intensity of each gene was calculated as follows: (Row signal intensity/75th percentile of each array) × 2,725. The average of the 75th percentiles from all of the arrays was 2,725.

## Additional file

## Electronic supplementary material

Additional file 1: Figure S1.: Expression of the HMW allergens in rice seeds. Expression levels of the HMW allergens during seed maturation stage (7 to 42 days after flowering) in endosperm and embryo were investigated by RiceXpro. Error bars represent standard deviation (*n* = 3). (PDF 113 KB)

Below are the links to the authors’ original submitted files for images.

Authors’ original file for figure 1

Authors’ original file for figure 2

Authors’ original file for figure 3

Authors’ original file for figure 4

Authors’ original file for figure 5

Authors’ original file for figure 6
