# Supplementary figures and images for: Generation of transgenic rice with reduced content of major and novel high molecular weight allergens
Source: Rice (N Y). 2014 Aug 29;7:19. doi: 10.1186/s12284-014-0019-0 (PMC4884044; doi:10.1186/s12284-014-0019-0)

Supplemental Figure 1

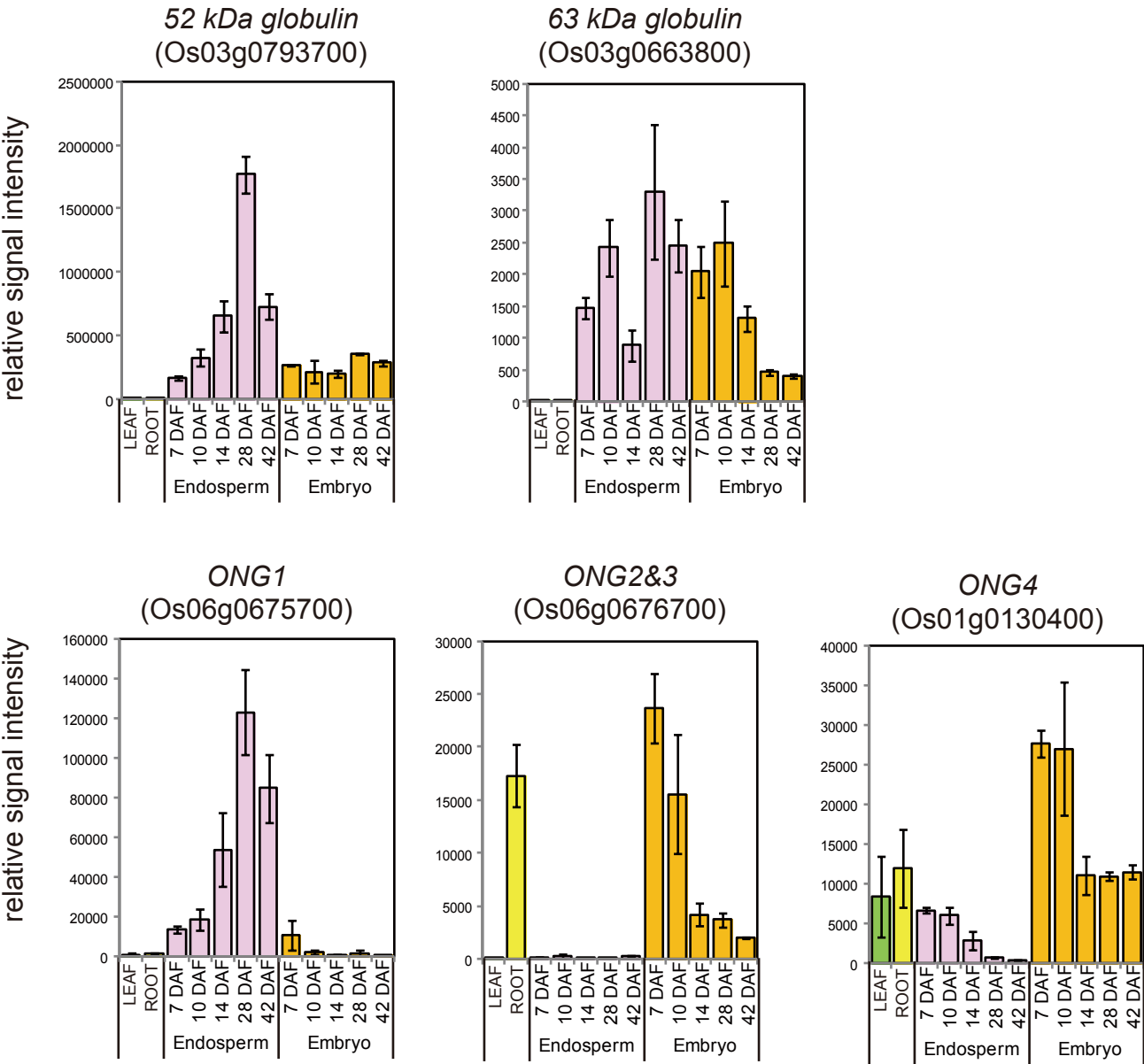

Supplement: Supplementary file 1 — Additional file 1: Figure S1.: Expression of the HMW allergens in rice seeds. Expression levels of the HMW allergens during seed maturation stage (7 to 42 days after flowering) in endosperm and embryo were investigated by RiceXpro. Error bars represent standard deviation (n = 3). (PDF 113 KB) [file 12284_2014_19_MOESM1_ESM.pdf]

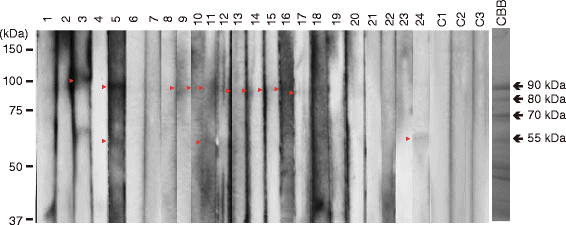

Supplement: Supplementary file 2 — Authors’ original file for figure 1 [file 12284_2014_19_MOESM2_ESM.gif]

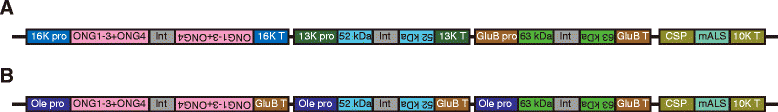

Supplement: Supplementary file 3 — Authors’ original file for figure 2 [file 12284_2014_19_MOESM3_ESM.gif]

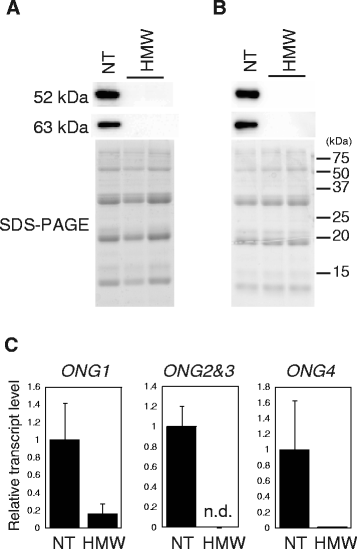

Supplement: Supplementary file 4 — Authors’ original file for figure 3 [file 12284_2014_19_MOESM4_ESM.gif]

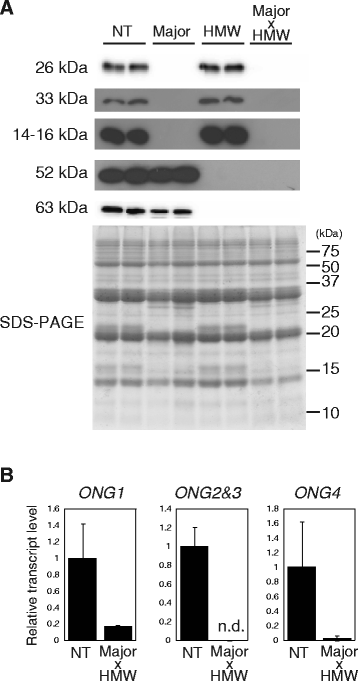

Supplement: Supplementary file 5 — Authors’ original file for figure 4 [file 12284_2014_19_MOESM5_ESM.gif]

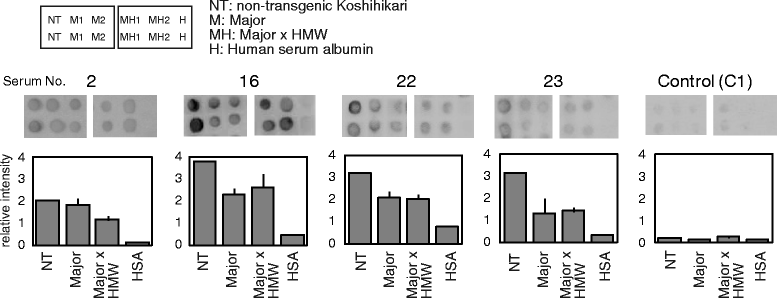

Supplement: Supplementary file 6 — Authors’ original file for figure 5 [file 12284_2014_19_MOESM6_ESM.gif]

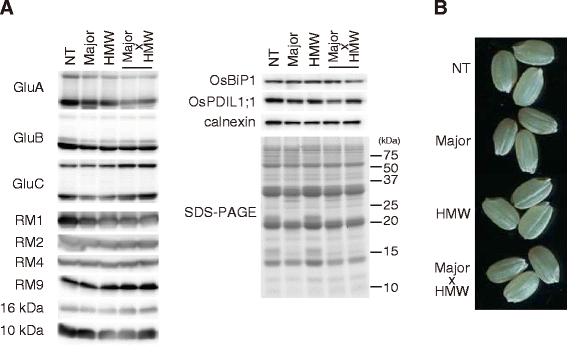

Supplement: Supplementary file 7 — Authors’ original file for figure 6 [file 12284_2014_19_MOESM7_ESM.gif]
